# Anterior chamber bacterial contamination in cataract surgery

**DOI:** 10.1186/1471-2415-14-57

**Published:** 2014-04-30

**Authors:** Jorge E Valdez-García, Alejandro Climent, Eduardo Chávez-Mondragón, Juan F Lozano-Ramírez

**Affiliations:** 1 Ophthalmology Research Chair, Escuela de Medicina y Ciencias de la Salud, Tecnológico de Monterrey, Av. Morones Prieto No. 3000 Pte. Col. Los Doctores, C.P, 64710 Monterrey, Nuevo León, México; 2 Instituto de Oftalmología, Fundación Conde de Valenciana IAP, Distrito Federal, Mexico

**Keywords:** Endophthalmitis, Cataract, Intraocular lens, Antibiotics, Phacoemulsification

## Abstract

**Background:**

The incidence of postoperative endophthalmitis has reduced during last several years to <0.01%; however, its associated complications continue to be devastating. Several sources of infection, including contamination by air, solutions, surgical instruments, intraocular lens, and wound leakage have been identified. The objective of this study was to evaluate the surgical technique, antibiotics, and asepsis that are used to reduce the risk of infection during cataract surgery.

**Methods:**

This was a transversal prospective study, in which 64 cataract surgeries were evaluated from 32 patients, with 1 month recovery time; and cultures from preoperative and postoperative aspirates were analyzed. Two groups were established based on whether preoperative antibiotics were given or not. The analysis employed descriptive statistics.

**Results:**

Of the 32 patients whose aspirates were obtained, three (9.37%) and 10 (31.25%) yielded positive cultures preoperative and postoperatively respectively. *Staphylococcus species* was the most common contaminating bacteria. The isolation of *Staphylococcus species may* indicate its potential as exogenous contaminant at time of wound closure. The cultures obtained from patients using preoperative antibiotics were positive for *S. aureus* in 10% (n = 2) of cases, and positive in 8.33% (n = 1) of cases not using antibiotics. The mean transoperative time with positive growth was 67 ± 17.8 minutes, and with negative growth was 76.3 ± 25.2 minutes. Two surgical techniques were evaluated: phacoemulsification and extracapsular extraction. The extracapsular technique showed a contamination rate of 33.33% (n = 8) compared to phacoemulsification with a rate of 25% (n = 2) (RR = 1.33).

**Conclusions:**

Common contaminating microorganisms included the *Staphylococcus* species, which was isolated from the eyelids and ocular annexes at the time of wound closure. The isolation of microorganisms postoperatively could have been influenced by the surgical technique used, the surgical time, and the use of antibiotics.

## Background

Although the incidence of postoperative endophthalmitis has generally been decreasing over the last few years, its associated complications continue to be devastating [1-3]. Several studies have identified different sources of infections, including trauma, eyelid margin, airborne contamination, solutions, surgical instruments, intraocular lenses, and wound leaks [1,2,4,5]. Nevertheless, in most cases, the ultimate source of the infection could not be identified, and the indigenous flora harbored in the eyelids and ocular annexes have been proposed to be responsible for the onset of bacterial endophthalmitis [5-7].

Recent reports, demonstrated that bacterial contamination of the anterior chamber during cataract surgery occurs in 20%–40% of cases [6,8-11]. Although the relationship between the presence of bacterial microorganisms and subsequent development of endophthalmitis has not been established, one report suggests that there is a relationship between the indigenous flora and the infecting organism in patients with endophthalmitis [12].

In the present study, we sampled aqueous humor in search of contaminating microorganisms at the time of cataract surgery. We obtained samples at the beginning of the surgical procedure, and at the time of wound closure following the cataract surgery; the patients were then followed for a period of at least one year. Some factors, such as type of surgery, duration, and the use of preoperative antibiotics, were investigated in order to establish a correlation between these factors and anterior chamber contamination, as these may be potential risk factors for the subsequent development of an infection.

## Methods

A prospective study was conducted among 32 patients undergoing consecutive cataract surgery giving a total of 64 surgeries with a 1-month recovery time between the surgical interventions from one eye to another eye in each patient. Patients with a history of previous ocular surgery, local or systemic infection, and ocular disease were excluded. This study was reviewed by the ethics committee of Fundación Conde de Valenciana IAP, and approved and written consent was obtained from all patients. In all cases, the same protocol was followed: All eyes were prepared for surgery using povidone–iodine solution for 5 minutes according to a standardized protocol [13]. At this point, 0.1–0.2 cc of aqueous humor was collected via paracentesis at the mid-lateral aspect using a 26 G needle, and cultured. On 20 patients, a topical antibiotic (tobramycin drops) was used just prior to beginning the surgery. At the end of the surgery, another 0.1–0.2 cc of aqueous humor was removed and cultured. Paraocular injections of dexamethasone phosphate (12 mg) were given.

Cataract surgery was performed using either the extracapsular extraction or phacoemulsification technique. All surgeries were performed using a fornix-based conjunctiva flap. A posterior chamber intraocular lens was inserted. Only one surgeon performed all of the surgeries.

Culture media for all cases consisted of blood agar, Thayer–Martin, thioglycolate broth, chocolate agar, tellurite agar, and mannitol salt agar. Cultures were incubated according to each medium used and held up to two weeks to allow for fungus and anaerobe growth. The identification of microorganisms was done basing on the standard microbiological procedures as previously described [14] by experienced laboratory personnel.

Follow-up visits consisted of a complete ophthalmologic evaluation with refraction, slit-lamp examination, and dilated ophthalmoscopy. Postoperative follow-up was completed at days 1, 3, and 30 after surgery, and 3 months, 6 months, and 12 months after surgery.

## Results

Organisms were identified in both preoperative and postoperative fluid aspirates. Three of 32 (9.37%) cultures from the preoperative aspirates were positive; the microorganisms that were identified were *S. epidermidis*, *S. hominis,* and *Diphteroids.* In 10 of 32 (31.25%) patient postoperative fluids, the culture was positive; the identified microorganisms were *S. epidermidis, S. aureus*, and *S. hominis* (Table 1). Three (9.37%) cases showed microorganism growth in both aspirates (preoperative and postoperative).

**Table 1:** Recovery of bacteria from the anterior chamber preoperatively and postoperatively

**Type of culture**	**No.**	**Positive cultures**	**%**
**Preoperative**	**32**	**3**	**9.37**
*S. epidermidis*		1	
*Diphtheroids (corynebacterium)*		1	
*S. hominis*		1	
**Postoperative**	**32**	**10**	**31.25**
*S. epidermidis*		6	
*S. aureus*		3	
*S. hominis*		1	

The patients were divided into two groups; patients in the first group (n = 20) were given preoperative antibiotics, where two patients (10%) yielded a positive culture. In the second group (n = 12), preoperative antibiotics were not used, and only one patient yielded a positive culture (RR = 1.20) (Table 2). A correlation between the organisms identified from the preoperative aspirates and those evident in the postoperative fluid was apparent in only one (3.12%) patient.

**Table 2:** Antibiotics exposure versus preoperative cultures

	**Preoperative culture**		
	**RR = 1.20**	**Positive**	**Negative**
Antibiotic exposure	Yes	2 (10%)	18 (90%)
	No	1 (8.33%)	11 (91.67%)

The mean transoperative (surgical) time for those with a positive culture was 67 ± 17.8 minutes, and the transoperative time for those with a negative culture was 76.3 ± 25.2 minutes.

The patients were followed at the indicated intervals, and none developed endophthalmitis. Two different cataract surgery techniques were evaluated: phacoemulsification and extracapsular extraction. The extracapsular technique showed a higher incidence of contamination with eight (33.3%) positive postoperative cultures and the phacoemulsification had two (25%) positive postoperative cultures (RR = 1.33) (Table 3).

**Table 3:** Association of type of surgery with positive cultures preoperatively and postoperatively

**Type of surgery**	**Positive culture (Preoperatively)**	**Positive culture (Postoperatively)**
Extracapsular Extraction (75%) (n = 24)	3 (12.5%)	8 (33.33%)
Phacoemulsification (25%) (n = 8)	0 (0%)	2 (25%)

## Discussion

It is interesting to highlight that the higher probability of a positive bacterial culture was observed among patients who used preoperative antibiotics when compared to those who did not use the antibiotics; this was an unexpected outcome.

While none of our patients developed endophthalmitis, approximately 0.1%–0.18% can develop endophthalmitis [15]. Several groups have documented bacteria in the aqueous humor of 20%–40% of eyes immediately following cataract surgery [8-10].

In order to minimize the risk of bacterial contamination of the aqueous humor and the development of endophthalmitis, the indigenous flora of the eye, lids and lashes can be reduced preoperatively by intracameral antibiotics [16,17]. The use of preoperative antibiotics to reduce the number and species of bacteri^a^ may be a possible solution [17]. An evidence-based research study showed that the recommended best practice, when examining all of the methods performed at the time of surgery, is the use of preoperative povidone–iodine with a B-II [17]. However, more studies regarding the role of antibiotics in the modification of indigenous flora are necessary.

In the present study, we were unable to show a significant reduction in positive postoperative cultures among those patients in which preoperative antibiotics were used (RR = 1.20). Some authors have shown a decrease in intraoperative contamination, which ultimately reduces a possibility of developing endophthalmitis [17].

The contaminating microorganisms isolated from the anterior chamber at the time of wound closure in the present study correlate with those isolated from the eyelids and ocular annexes of a similar population [7]. In our opinion, these results support the hypothesis that the aqueous humor in a significant number of patients is contaminated with organisms, and that this is a common cause of endophthalmitis [2,3]. Even more, the presence of *S. aureus* in postoperative cultures can be an indicator of exogenous contamination at the time of wound closure, in this study, but contrary to this, the indigenous flora of the eyelid margin and ocular surface has been proposed as a probable and major source of infection in postoperative bacterial endophthalmitis [7].

A lower incidence rate of contamination was observed in the phacoemulsification technique group when compared to the extracapsular extraction group, and this raised the concern that other factors play a role in the development of endophthalmitis. Some authors have proposed that minimizing the extraocular fluid entry into the eye during surgery may contribute to decreasing the incidence of contamination [5,6]. Another theory is that there is a relationship between the eye’s exposure time to the environment and the incidence of bacterial contamination. However, the present study showed that there was no difference in the mean duration of the surgery among those with positive cultures and those with negative cultures at the end of surgery. Moreover, it has also been proposed that the amount of time the eye is exposed to the environment be minimized, so as to reduce contamination [12]. A possibility to consider is the wound size, this could be a risk factor for contamination during extracapsular extraction technique.

In our opinion, the findings in the present study support the proposal that anterior chamber contamination can occur with the first exchange of intraocular and periocular fluid. Our evidence supports the idea that the anterior chamber contamination that occurs during cataract surgery might be more related to extraocular fluid interchange than to the duration of surgery.

Although it has been reported that bacterial endophthalmitis occurred within 7 days in 77% of cases, and that all cases occurred within 32 days, we decided to extend the follow-up period to one year, which was due to the observation of a case of endophthalmitis that occurred seven months after the extracapsular cataract extraction [18].

We should also recognize intraocular lenses as a potential source of contamination; polymethylmethacrylate (PMMA) intraocular lenses have a higher incidence rate of bacterial endophthalmitis [19]. However, our study did not evaluate intraocular lenses. As well, a limitation to our study was only taking anterior chamber samples and not from ocular adnexa for culture, in order to identify correlation between the anterior chamber isolates and those found in the adnexa.

## Conclusions

Anterior chamber contamination may regularly occur during cataract surgery, with *Staphylococcus* species isolated notably at the time of wound closure. The type of indigenous flora, the extraocular fluid’s entry into the anterior chamber, and the amount of fluid in the chamber, may influence its incidence. Further studies are needed to elucidate which factors, including inoculum size and type, bacterial pathogenicity, and host defense mechanism and status, are involved in bacterial contamination and the development of endophthalmitis, so as to better understand the pathogenesis of both of these phenomena.

## Competing interests

None of the authors has personal, financial, or non-financial competing interests that could influence or affect the interpretation or presentation of the information.

## Authors’ contributions

JEVG has made substantial contributions to the study’s conception and design, revising the manuscript critically for important intellectual content, and provided final approval of the version to be published. AC made substantial contributions to the study’s conception and design, the acquisition of data, and the analysis of data. ECM ensured that the questions related to the accuracy or integrity of any part of the work were appropriately investigated and resolved, and also revised the manuscript critically for important intellectual content. JFLR performed data acquisition, interpreted the data, drafted the manuscript, and prepared it for submission. Authors internally funded the research.

## Pre-publication history

The pre-publication history for this paper can be accessed here:

http://www.biomedcentral.com/1471-2415/14/57/prepub
